# Physiology

**Published:** 1861-09

**Authors:** S. Weir Mitchell

**Affiliations:** Lecturer on Physiology in the Phila. Med. Association


### PHYSIOLOGY.

BY S. WEIR MITCHELL, M.D.,

LECTURER ON PHYSIOLOGY IN THE PHILA. MED. ASSOCIATION.

I.—*A Case of Preternatural Anus, with Contributions to the Physiology of Digestion.* By W. Braune.

W. Braune has had the opportunity of examining the phenomena of digestion in a fistula of the ileum, or rather a preternatural anus, situated about twenty-four centimetres (rather more than nine inches) above the ileo-cœcal valve. The fistula was the consequence of a neglected or maltreated hernia near the umbilicus, in a woman, aged forty-nine. The communication between the upper and lower portion of the intestinal tract was completely closed. The chyme issuing from the fistulous opening after meals was, in general, rather thin, had an acid smell, and a distinctly acid reaction, while the mucous membrane of the ileum turned the red litmus-paper blue. During fasting the reaction of the chyme was neutral. The chyme contained no cholepyrrhin, but a considerable quantity of biliary acids; there was no proof of unchanged casein, while Busch, in his case of fistula of the jejunum, distinctly noticed it; sugar was absent; uric acid was not found. The aqueous extract of the chyme exercised no influence on coagulated albumen. The excretions from the anus consisted only of mucus and epithelium, occurring about once in four weeks, and emitting a very fetid smell. With regard to the time elapsed between the ingestion of food and the appearance of the remains in the chyme, the first portion of meat was recognized after three hours, and no more traces were found after six hours from the meal; the skins of grapes, when taken fasting, were passed after three hours and five minutes; when taken with other food, after three hours and three-quarters. In Busch's case, where the fistula was in the commencement of the jejunum, the first portions of meat appeared from between twenty-two to thirty minutes after the commencement of the eating; the passage from the jejunum to the lower portion of the ileum appears therefore to require between two hours and three-quarters and five hours and a half. The *temperature* in the intestine was always rather higher than in the axilla. After meals there was constantly a perceptible increase of temperature as well in the intestine as also in the axilla, the greatest increase amounting to 0·90 F.—(*Virchow's Archiv*, vol. xix. p. 470, 1860; *The British and Foreign Medico-Chirurgical Review*, July, 1861.)

II.—*On the Influence of Change of Atmospheric Pressure on the Human Organism.* By R. VON VIVENOT, Jun.

Vivenot has examined the phenomena observable in man when exposed to sudden alterations of atmospheric pressure. Rapid and considerable *diminution* of pressure—as experienced in the ascent in an air-balloon to the height of about 18,000 feet above the sea, or by the climbing of mountains of a similar elevation, connected with a reduction of pressure to one-half of what we are generally exposed to—is found to cause: 1. Increased evaporation from the lungs and surface of the body. 2. Increased oxidation, in consequence of the loss of warmth occasioned by the augmented evaporation. 3. Increased frequency of respiration and pulsation, the former being induced as well by the smaller amount of oxygen contained in a certain volume of air, as also by the greater amount required in the formation of heat. 4. Congestion of blood toward the peripheric parts, and in consequence dilatation and even rupture of the blood-vessels. 5. Diminished approximation of the corresponding portions of joints. 6. Diminished secretion of urine, as an effect of the increased evaporation. The observations on *increased* atmospheric pressure have been made in Tabarié's air-compressing apparatus, as it is used for therapeutic purposes at Nice, Montpellier, and other places, under the name "*bain d'air comprimé*." The author's inferences are based on researches made at Nice on himself and four other male individuals. For the description of the apparatus we refer to the essay itself. The experiments are made on eight successive days, between the hours of twelve at noon and two P.M., before the principal meal, the mean temperature being 11° Cent., (51·8° Fah.,) the atmospheric pressure in the apparatus amounting to $1\frac{1}{2}$ atmospheres, (925·04 millimetres* mercury.) The effects of the compressed air were: 1. A decided decrease in the frequency of pulsation: the greatest decrease being eighteen beats per minute, the average ten beats. 2. Diminution of the number of respiratory movements, amounting in the mean to 1·1 per minute. The influence, as well on the pulse as also on the respiration, lasted beyond the time of the experiment; it seemed to be

* A millimetre = 0·03937 inch, or 0·47 line.

greater where the frequency of pulse and respiratory movements were morbidly increased. 3. Diminished evaporation from the surface of the skin and lungs. 4. Increased secretion of urine. 5. Repulsion of the blood from the periphery, especially checking of congestive conditions in the periphery. The author further expresses the opinion, that the slight variations of atmospheric pressure occurring under usual circumstances, with change of weather, are of little influence on our feeling of health and strength, and that the phenomena frequently ascribed to these slight changes are due to other influences, as temperature, moisture, and winds.—(*Virchow's Archiv*, vol. xix. p. 492, 1860; *The British and Foreign Medico-Chirurgical Review.*)

III.—*Contribution to the Physiology of the Liver; Influence of Alkalies.* By F. W. PAVY.

Pavy has examined the influence of injections into the jugular vein of carbonate of soda in solution, on the appearance of diabetes after section of the sympathetic nerve of the neck. He injected in other experiments solutions of carbonate of soda into the stomach and into the portal vein. The principal inferences of the author are: 1. The introduction of carbonate of soda into the circulation prevents the production of saccharine urine, after lesions of the sympathetic nerve, otherwise occasioning it. 2. Carbonate of soda, injected into the general venous system, does not prevent the urine from becoming saccharine after the destruction of life, when the circulation is kept up artificially; but injected into the portal system, so that all may pass into the liver, it has the effect of keeping the urine entirely free from sugar. 3. Carbonate of soda, injected into the portal system during life, causes a rapid disappearance of the hepatine from the liver, without any sign of the production of sugar. 4. In the disappearance of hepatine under the influence of carbonate of soda, the hepatine is not concealed, nor transformed into sugar, nor destroyed by any direct chemical power; but may be transmuted by a process analogous to catalysis, into another substance which, as yet, has escaped discovery. 5. Carbonate of soda, injected into the liver after death, does not effect a disappearance of hepatine; but even when injected in moderate quantity, holds the saccharine metamorphosis completely in check. The author further suggests that there is a close connection between the disappearance of hepatine, the production of fat, and the state of the bile.—(*Proceedings of the Royal Society*, vol. xi. p. 90, 1861; *The British and Foreign Medico-Chirurgical Review.*)

IV.—*On the Influence of the Pneumogastric and the Superior Laryngeal Nerves on the Movements of the Diaphragm.* By J. ROSENTHAL.

Rosenthal communicates to the Academy of France that he has found that irritation of the central portion of dissected superior laryngeal nerve influences the action of the diaphragm. The manner, however, in which irritation of the superior laryngeal nerve acts is different from that manifested by irritation of central end of the pneumogastric nerve below the giving off of the superior laryngeal; while moderate irritation of the pneumogastric nerve arrests the diaphragm in the state of contraction, that of the superior laryngeal arrests it in the state of relaxation; while very weak induced currents applied to the pneumogastric cause a remarkable acceleration of the movements of the diaphragm, the same applied to the superior laryngeal nerve cause retardation. Rosenthal regards the superior laryngeal nerve as an "inhibitory nerve."—(*Compt. Rend.*, vol. lii. p. 754, 1861; *The British and Foreign Medico-Chirurgical Review*, July, 1861.)

